# Rethinking the utility of the Five Domains model

**DOI:** 10.1017/awf.2023.84

**Published:** 2023-09-27

**Authors:** Jordan O Hampton, Lauren M Hemsworth, Paul H Hemsworth, Timothy H Hyndman, Peter Sandøe

**Affiliations:** 1 Animal Welfare Science Centre, Faculty of Science, University of Melbourne, Parkville, VIC 3010, Australia; 2 Harry Butler Institute, Murdoch University, 90 South Street, Murdoch, WA 6150, Australia; 3 School of Veterinary Medicine, Murdoch University, 90 South Street, Murdoch, WA 6150, Australia; 4 Department of Food and Resource Economics and Department of Veterinary and Animal Sciences, University of Copenhagen, Rolighedsvej 25, DK-1958, Frederiksberg, Denmark

**Keywords:** aggregation, animal welfare, objectivity, public relations, scientific method, wildlife

## Abstract

The Five Domains model is influential in contemporary studies of animal welfare. It was originally presented as a conceptual model to understand the types of impact that procedures may impose on experimental animals. Its application has since broadened to cover a wide range of animal species and forms of animal use. However, it has also increasingly been applied as an animal welfare assessment tool, which is the focus of this paper. Several critical limitations associated with this approach have not been widely acknowledged, including that: (1) it relies upon expert or stakeholder opinion, with little transparency around the selection of these individuals; (2) quantitative scoring is typically attempted despite the absence of clear principles for aggregation of welfare measures and few attempts to account for uncertainty; (3) there have been few efforts to measure the repeatability of findings; and (4) it does not consider indirect and unintentional impacts such as those imposed on non-target animals. These deficiencies lead to concerns surrounding testability, repeatability and the potential for manipulation. We provide suggestions for refinement of how the Five Domains model is applied to partially address these limitations. We argue that the Five Domains model is useful for systematic consideration of all sources of possible welfare compromise and enhancement, but is not, in its current state, fit-for-purpose as an assessment tool. We argue for wider acknowledgment of the operational limits of using the model as an assessment tool, prioritisation of the studies needed for its validation, and encourage improvements to this approach.

## Introduction

Animal welfare science remains a young and dynamic discipline. Approaches to conceptualising and measuring animal welfare have evolved considerably since the modern discussion of animal welfare began in the 1960s (Broom 2011). Like in any other branch of science, progress and evolution in our understanding of animal welfare rely upon challenge, debate and argumentation. However, there are suggestions that progress has been slowing in several major fields of science, becoming ‘less disruptive’ in recent decades. One recent meta-analysis showed that newer papers are increasingly less likely to break with the past in ways that push science in new directions (Park *et al.* 2023). This trend should be counteracted in animal welfare science.

In this paper, we highlight one animal welfare paradigm that, despite being nearly 30 years old, and widely used in many (but not all) global regions, has not been robustly challenged: the Five Domains model. We specifically explore the limitations of the Five Domains model when used as an animal welfare assessment tool. We provide a brief review of how the Five Domains has silently evolved from a conceptual model into an assessment tool. In doing so, we aim to provide a constructive review of the limitations of this approach, provide examples of misuse, make suggestions for refinement, and discuss alternative approaches. We consider the possibility that the absence of ‘disruptive’ studies challenging this paradigm may be slowing the evolution of the animal welfare discipline. We begin with a brief review of when, how and where the model has been used.

## The history of the Five Domains model

The Five Domains model was proposed approximately 30 years ago as a conceptual framework to simplify animal welfare considerations for research animals (Mellor & Reid 1994). It was built on the foundations of the Brambell Report (Brambell 1965) and the Five Freedoms model (Farm Animal Welfare Committee 1979). The model was initially created to assess the impact of a proposed animal experiment or usage by considering all sources of possible welfare compromise, that is, negative welfare (at least on animals directly and intentionally impacted by humans).

The Five Domains model is based on the affective state (or feelings)-based conception of animal welfare called ‘hedonism’ in the philosophical literature (Appleby & Sandøe 2002), which is one of the mainstream views of animal welfare (Beausoleil *et al.* 2018), and is accounted for in a transparent way by the authors of the model (Mellor *et al.* 2020).

The model proposes four physical/functional domains (nutrition, environment, health and behaviour (recently renamed ‘behavioural interaction’ [Mellor *et al.* 2020]) and the fifth domain is the so-called mental state. The basic idea is that the welfare state reflects the sum of the animal’s mental experiences (Harvey *et al.* 2022). Hence, the welfare status of an animal is a direct function of the feelings of the animal: the mental domain. The Five Domains model then, in turn, interprets the experiences of animals as the function of four other aspects or ‘domains’ of animals’ lives: their nutritional state, the environment in which they live, their physical health, and their behavioural opportunities (Mellor & Beausoleil 2015). In short, the model says that animals’ welfare is determined by the quality of their experiences, and our best evidence regarding the quality of their experiences comes from the four other domains.

Subsequently updated, the model has had various manifestations since its first conceptualisation (Mellor & Beausoleil 2015; Mellor 2017; Mellor *et al.* 2020) and has recently been extended to incorporate positive welfare states (Mellor & Beausoleil 2015) and human-animal interactions (Mellor *et al.* 2020).

### From a conceptual framework to a measurement tool

Conceptual use of the Five Domains model aligns with what it was designed for. In this context, the outputs of the model are used to identify negative animal welfare impacts (risks) and positive animal welfare impacts (enhancements) associated with any animal manipulation activity. This conceptual way of applying the model has been framed as “risk assessment” (Sherwen *et al.* 2018) when the aim is to identify knowledge gaps and research priorities relating to negative welfare. However, the term “hazard identification” may be a more appropriate term in light of the risk analysis framework (EFSA Panel on Animal Health and Welfare 2012). Conceptual use of the model has also been framed as “identifying opportunities to promote positive welfare” (Kells 2021) when the focus is on positive welfare. Put another way, using the model as a conceptual tool can be thought of as a “focussing device” (Mellor 2017) for animal welfare discussions. Importantly, conceptual applications of the model do not attempt to quantify or rank welfare outcomes. But how do we go from *thinking about* different contributors to animal welfare to scoring them, ranking them or comparing them? This is much less clear.

This second type of application is importantly different from conceptual studies (although the distinction is not always recognised by the authors of such studies). In this case, the model is used as something it was not designed to be: a (purportedly) quantitative, scientifically robust animal welfare assessment tool that is often used to rank different techniques (Sharp & Saunders 2011). Over the past two decades an increasing number of authors and organisations have used the Five Domains model in this way. But, before this is discussed, it is necessary to reflect on *how* the model has been adapted into an assessment tool that is then used to assess animal welfare.

### Generation of scores in assessments

Although there is considerable variation in the methods used by studies attempting to deploy the Five Domains model as a quantitative assessment tool, there are some common features (Mellor 2017). Mellor (2017) provides an instructive account of the operational details of using the Five Domains model as an animal welfare assessment tool, and an earlier account is provided by Sharp and Saunders (2011). First, an individual possible intervention (or even an individual animal) (Littlewood & Mellor 2016) or group of interventions (e.g. 14 rodent control methods) (De Ruyver *et al.* 2023) is defined for assessment. Second, a panel of experts (animal welfare scientists) or stakeholders (technicians, community members or representatives of advocacy groups) are assembled. Third, background/summary literature summaries are provided to each panel member by a convener. Fourth, the panel are asked to provide numerical/categorical scores for each domain, each technique and, in some cases, for more than one phase of the intervention studied, e.g. (a) prior to death, and (b) mode of death (De Ruyver *et al.* 2023). These scores are meant to reflect the magnitude of negative (and recently, positive) feelings an animal might experience in each of the Five Domains, both prior to death and via their mode of death (if a lethal method is used) (Baker *et al.* 2016). These scores are typically given on an ordinal scale of least-to-most suffering, e.g. 1–8 (De Ruyver *et al.* 2023), 0–5 (Hampton *et al.* 2016a) or A–D (Littlewood & Mellor 2016) in a per-category scoring system. The outputs are then used to rank the techniques assessed, e.g. a score of 4D (4 on a 0–5 scale for suffering prior to death, and D on a scale of A–H for suffering due to mode of death) for ground-based chest shooting of wild dromedary camels (*Camelus dromedarius*) (Hampton *et al.* 2016a). Such outputs are described as “systematic, holistic, data-based assessments” (Beausoleil *et al.* 2022).

### Contemporary applications

Since the 1990s, the Five Domains model has been applied to a variety of animal groups impacted by human activities. The model has been particularly widely used by investigators in New Zealand, Australia, and Europe (Table 1). However, the Five Domains model has far from a global monopoly on animal welfare assessment, with comparatively little use in the global regions of North and South America, Asia and Africa (Table 1). Today, the Five Domains model is being used more and more widely to understand anthropogenic effects on the welfare of a wide range of animals, including research animals, wildlife, livestock, and companion animals (Table 1).

**Table 1.** Methodological details of peer-reviewed studies that have used the Five Domains model as an animal welfare assessment and ranking tool. NR = not reported. NA = not applicable. SOP = standard operating procedure.

Year	Country	Target species	Technique/s assessed	# of panel members	Panel inclusion criteria	Scoring system	Method of information retrieval	Agreement method	Measures of uncertainty	Measures of repeatability	Citation
2016	UK	1. European rabbit (*Oryctolagus cuniculus*), 2. European mole (*Talpa europaea*), 3. Carrion crow (*Corvus corone*)	9 lethal and non-lethal control methods	1	NR	Part A: 1–8, Part B: A–H*	Development of SOPs	NA	NR	NR	(Baker *et al.* 2016)
2016	New Zealand	Brushtail possum (*Trichosurus vulpecula*)	7 poisons	6	“Scientists”	No, Mild, Moderate, Severe or Extreme	“Relevant literature”	Median scores: 1. Confidential individual scores, 2. Meeting	0–3	NR	(Beausoleil *et al.* 2016)
2016	Australia	Dromedary camel (*Camelus dromedarius*)	3 lethal control methods	NR	NR	Part A: 0–5, Part B: A–H*	NR	NR	NR	NR	(Hampton *et al.* 2016a)
2018	Australia	Multiple zoo species	Captive animal husbandry	NR	“Experienced zoo Personnel”	0–2 (‘poor’ to ‘good’)	NR	NR	NR	NR	(Sherwen *et al.* 2018)
2019	Australia and South Africa	Multiple wildlife and domestic species	Livestock guardian animals	13	“Experts”	Part A: 1–8, Part B: A–H*	NR	NR	NR	NR	(Allen *et al.* 2019)
2022	UK	Norway rat (*Rattus norvegicus*)	6 lethal control methods	15	“Experts”	Part A: 0–5, Part B: A–H*	“Best practice SOP + background reading”	“Consensus”	0–3	NR	(Baker *et al.* 2022)
2023	Belgium	1. House mouse (*Mus musculus*), 2. Norway rat, 3. Black rat (*Rattus rattus*)	14 lethal control methods	8	“Based on expertise”	Part A: 1–8, Part B: A–H*	“Background reference document”	1. Confidential individual scores, 2. Online focus group	Option not to score some techniques	NR	(De Ruyver *et al.* 2023)

*Two scores reported for some lethal wildlife control techniques: Part A = before death, and Part B = mode of death.

#### Animals used in research

The Five Domains model was first used in a regulatory context to systematically assess the welfare impacts of animal research activities in New Zealand (Mellor & Beausoleil 2015). For example, integer scores are awarded out of five for each of the four physiological domains (A, B, C and D) and the aggregate of this score (a composite score) is then used to appraise the harm that is imposed on the research animals. It has subsequently been adopted by several animal research institutions, but this type of use is rarely published.

#### Free-ranging wildlife

The Five Domains has seen perhaps the widest uptake in the field of wildlife management. It has been adopted for over ten years to assess practices used to kill and remove introduced wildlife species in Australia (Sharp & Saunders 2011; Hampton *et al.* 2016a; Harvey *et al.* 2020, 2021, 2023). It has since been applied to introduced wildlife in New Zealand (Beausoleil *et al.* 2016), nuisance (‘pest’) native wildlife species in the United Kingdom (Baker *et al.* 2016, 2022) and other countries such as Belgium (De Ruyver *et al.* 2023). The model has also been used as an assessment and ranking tool to examine internationally practiced management actions such as the use of guardian animals to protect livestock from attacks by wild predators (Allen *et al.* 2019). More recently, the model has been applied to inform management actions in emergency scenarios involving native conserved wildlife, such as whale strandings in New Zealand (Boys *et al.* 2022a,b).

#### Zoos and aquaria

There is growing use of the Five Domains for captive wildlife (Clegg *et al.* 2015; Sherwen *et al.* 2018), with significant uptake among zoos (Kagan *et al.* 2015; Ward *et al.* 2020). In fact, the World Association of Zoos and Aquaria’s (WAZA) animal welfare strategy recommends that zoos and aquaria apply the Five Domains model to assess animal welfare (Mellor *et al.* 2015).

#### Companion animals and horses

There have been a limited number of studies to apply the Five Domains model to the welfare of pet dogs (*Canis familiaris*) (Littlewood & Mellor 2016; Ledger & Mellor 2018). Several recent studies have applied the Five Domains model to the welfare of domestic horses (*Equus caballus*) (McGreevy *et al.* 2018; Mellor & Burns 2020; Fletcher *et al.* 2021; Harvey *et al.* 2022).

#### Livestock

There have been few published studies in which livestock welfare has been discussed in relation to the Five Domains model. The few studies that have adopted the model have done so in a conceptual way, to identify animal welfare indicators and measurement options for assessment tools and audits. Such studies have applied the model to sheep (*Ovis aries*) farming (Fogarty *et al.* 2019; Hernandez *et al.* 2020) and for livestock production generally (Grandin 2022), and to identify opportunities for positive welfare enhancement (Kells 2021; Johnson *et al.* 2022).

## Problems with use of the Five Domains model as a welfare assessment tool

What began as a way of conceptualising all of the complex inputs that might affect animal welfare, clearly rooted in an affective state viewpoint has, as we have seen, been developed into an *assessment* tool producing scores purporting to compare and rank different human interventions on the welfare of a range of animals.

However, we think there are several critical limitations of this kind of use of the Five Domains approach. These problems have not been widely acknowledged by the animal welfare community, and the growing use of the model as an assessment tool (Table 1) has gone largely unchallenged in the animal welfare literature, with few (if any) published studies critiquing these findings. We highlight some of the key methodological details of studies that have used the Five Domains model as an animal welfare assessment and ranking tool in Table 1. In populating this table, we searched for peer-reviewed studies that have generated assessment scores using the model. We did not include non-peer-reviewed studies, e.g. Sharp and Saunders (2011), nor those peer-reviewed studies that applied the model to hypothetical animals or scenarios, e.g. Littlewood and Mellor (2016), and Harvey *et al.* (2020). We describe some of the critical problems with such studies below.

### Subjective experiences to objective scores

One of the central challenges of animal welfare science is how to translate something fundamentally subjective, animals’ feelings, into objective terms. Simply, there is no direct way to measure the experiences or feelings of animals (Browning 2022b). One strength of the Five Domains model is that it starts with a clear and transparent notion of animal welfare, and the developers of the model recognise that this notion is subjective and therefore cannot be measured directly (Mellor 2016). So, how are animal welfare inferences made? This is a general challenge of quantitative animal welfare assessment – providing an objective view of a subjective state (Fraser & Duncan 1998).

Therefore, even if the welfare of animals is determined by the overall quality of their experiences, the study of their welfare has to be focused on detectable indicators of these experiences (Fraser 2008). If mental experiences such as pain, breathlessness and fear cannot be measured directly, they must be cautiously extrapolated from observable indicators of the animal’s physical or physiological state or its behaviour. This task is not straightforward (Browning 2022a). How these judgements are made, who is qualified to make them, how their accompanying scores are reached, and what evidence is considered in reaching these conclusions, are just some of the obstacles in the way of making defensible conclusions.

A common answer to this problem is to gather a panel to make the assessment.

### Selection of panel members

The assessments generated from the Five Domains model are derived through the use of panels and suffer from the problems inherent to the use of expert opinion or ‘eminence’ (Hampton *et al.* 2016b). This is of particular concern for contexts in which there are gaps in scientific understanding, and model outputs may thereby fail to be evidence-based (Baker *et al.* 2016). Ten obvious questions here are: (1) *who is selected* to sit on a Five Domains panel?; (2) *who decides* on panel member selection?; (3) what *criteria* are used to include or exclude potential panel members?; (4) *how many* panel members are selected?; (5) how will the *panel members interact*?; (6) will a *consensus* be sought?; (7) if so, *how* will a consensus be reached?; (8) what happens *if a consensus cannot be reached*?; (9) *who will scrutinise* the panel’s decisions?; and (10) how is *impartiality* assessed? Much hinges on the answers to these questions.

Humans are prone to confirmation and disconfirmation biases (Nickerson 1998) – we interpret evidence to support conclusions we want to reach, and we see what we want to see. These biases certainly extend to animal welfare questions (Buddle *et al.* 2018), and the Five Domains model is not unique in suffering from these problems; they apply to any use of expert panels. So, the panel approach introduces considerable subjectivity to the process. For instance, some people sympathise more with certain animals compared to others. Likewise, some people start the process with an attachment to one or another of the proposed methods (e.g. zookeepers or other zoo employees may be inclined to favour existing zoo husbandry practices) (Sherwen *et al.* 2018). Conversely, panel members familiar with and supportive of existing practices may be unduly critical of newly developed or newly proposed alternative practices or technologies (Johnson *et al.* 2019). There are also gender and ethnic disadvantages to consider (e.g. men ‘silencing’ women) (Shpungin *et al.* 2012), as well as the rarely considered benefits of including indigenous knowledge and perspectives (Normyle *et al*. 2022).

Few of the studies that have used the Five Domains model as an assessment and ranking tool have reported how panel members have been selected (Table 1). This raises the important question of whether panel members are selected because of their expertise in animal welfare science, special knowledge/insight, relevance as a stakeholder, desire to volunteer, ideological alignment with colleagues, affirmative action criteria, personal relationship to the panel convenor, or something else. There is a serious concern that unstated conflicts of interest will be present in this environment, with a prime example being the possibility of a panel member that is funded by an industry and who demonstrates bias towards the funding agencies’ interests (Van der Schot & Phillips 2013).

### Experts or stakeholders?

It is often not clear whether panel members are selected as ‘experts’ or ‘stakeholders.’ Some published studies have specified the need for diverse and non-scientific backgrounds for panel members: “there would be merit in engaging panels or consultative networks with wide expertise and experience” (Mellor & Beausoleil 2015). Mellor *et al.* (2020) state that “Any assumption of the occurrence of negative affects must be supported by directly observed animal-based physical, physiological, clinical and/or behavioural evidence”. The same authors go on to say, “This is equally the case for the presence of opportunities for animals to engage in rewarding behaviours.” Clearly, there must be evidence, usually behavioural, that any such opportunities are actually used before their potential welfare-enhancing impacts could be considered. Only then can inferences be made about any aligned negative or positive effects. Finally, Mellor *et al.* (2020) posit that “This emphasises the general point that objective animal-based evidence (Domains 1 to 4) must form the foundations of any inferences about welfare-relevant affects (Domain 5).”

Thus, the authors that developed and refined the conceptual model seem to be recommending that this evidence should be utilised by whoever is doing the assessment (individual or panel). So then why specify diverse and non-scientific backgrounds for panel members? The issue of scientific literacy must then be addressed, i.e. raising the question of whether non-expert panel members can comprehend the relevant evidence. This leads to questions regarding exactly what the criteria are for choosing (‘inclusion criteria’) or not choosing (‘exclusion criteria’) potential panel members. These are important factors to consider when regarding the findings of any panel, e.g. juries (Cullen & Monds 2020), but are rarely reported in Five Domains assessment studies (Table 1).

### Consensus or majority, confidential or discussive?

The next key question is *how* panel members are giving their views and how collective decision-making is achieved. Views may be given individually (this may be confidential, anonymous or blinded), or in a group process. There are merits to the former, as used by De Ruyver *et al.* (2023) to avoid “groupthink” (Resnik & Smith 2020), a phenomenon that occurs when a group of individuals reaches a consensus without critical reasoning or evaluation of the consequences or alternatives. Groupthink is based on a common desire not to upset the balance of a group of people. Not all studies that have used the Five Domains model as an assessment and ranking tool have reported which approach they have used (Table 1).

For consensus methods, there is the issue of disproportionate influence of dominant persons (Gavrilets *et al.* 2016). In other words, all conclusions reached are stated to be the product of the deliberations of the panel members but may effectively reflect which panel members were most opinionated or domineering. Some Five Domains assessment studies have recognised this pitfall, and have instead required each panellist to independently generate scores, which are then discussed at a subsequent meeting of the panel, with final scores reported as average values (e.g. medians) (Beausoleil *et al.* 2016), rather than attempting to reach consensus.

### Use of published and quantitative research

For many published Five Domains assessment studies, there are scant details provided of how previously published research is used to reach scores. As can be seen in Table 1, details of literature searches are rarely reported in studies that use the Five Domains model as an assessment and ranking tool. Mellor *et al.* (2020) refer, somewhat obliquely, to “scientifically informed best judgement” in describing how this process occurs. This is sound advice, but it leaves a number of important details to the discretion of the panel: who collates the scientific evidence, what evidence is deemed to be relevant to the context (e.g. are studies from other species considered), and who reviews whether the collation is appropriate?

There is considerable variation in how each Five Domains assessment reviews and presents available evidence to panel members. McGreevy *et al.* (2018), for example, stated that “each context leader supplied an overview of the context, as well as annotated references, to support welfare assessments during the workshop. This was distributed to the panellists as a handbook.” Whereas, Sherwen *et al.* (2018) described their efforts to “systematically collect information from a team of experienced zoo personnel who included zookeepers, veterinarians, managers, and a welfare researcher/specialist to allow potential and/or current risks to animal welfare to be identified.” While these efforts are admirable, these processes more closely align with roundtable discussions than controlled scientific reviews.

### Repeatability

We are unaware of any studies that have tested the repeatability of Five Domains assessments. Reliability is a core tenet of the scientific method and is defined as the extent to which measures are repeatable and consistent, i.e. the similarity between repeated measurements of the same item (Windschnurer *et al.* 2008). This is a fundamental requirement of any reliable scientific measurement method.

Reliability for animal welfare assessments can be classified into interobserver and test-retest (or repeatability) reliability (Vaz *et al.* 2013). Interobserver reliability assesses the role that differences play among observers (Harley *et al.* 2021). Different, but similarly trained, observers should obtain the same results when assessing the same animals at the same time under the same circumstances but independently from each other (O’Callaghan *et al.* 2003). An interobserver reliability study of Five Domains panels would assess whether different panels performing assessments on an identical practice repeat the findings of a separate panel. Test-retest reliability characterises the consistency of the method over time and, thus, the repeatability of the results (Vaz *et al.* 2013). These measures have been quantified for other animal welfare assessment systems, e.g. Welfare Quality® (Friedrich *et al.* 2020), but never for the Five Domains, casting doubt over how reliable its outputs are.

### Aggregation

To be able to reach a conclusion about the net welfare outcome of a welfare assessment based on the Five Domains model, it is necessary to present a model on how to aggregate the values of the different criteria scored by a panel. However, impact categories are ordinal, and differences between pairs of adjacent categories may not be linearly related (Baker *et al.* 2016). According to the statement of the latest (2020) outline of the model, this ambition cannot be fulfilled due to the “limits imposed by an inability to determine the relative impacts of different affects when evaluating the notional overall negative–positive affective balance represented by QoL (quality of life), thereby precluding the possibility of elaborating an all-inclusive QoL metric” (Mellor *et al.* 2020).

Given these limitations, all that can effectively be done is to assess how different aspects of the four first domains contribute positively or negatively to the overall welfare, but not how the many positive and negative inputs add up in terms of the net welfare of the animals in question. However, since conclusions in terms of net welfare outcomes are actually reached, the way aggregation is undertaken should be made transparent. Therefore, it is fair to ask how the scores awarded for each domain are weighted when added to one another. Further, do any of the domain scores strongly correlate with one another? That is, does one domain score predict the fifth Domain of mental state in a similar way to another domain score, making one of the two domain scores redundant? In a later section, we discuss how alternative welfare assessment models tackle the problem of aggregation in more robust ways.

### Uncertainty

Conclusions reached through use of the Five Domains model are ultimately qualitative in nature but are commonly denoted by numerical scores (e.g. 3 out of 5). What is often lacking (aside from transparent explanation of how these scores are derived) is a measure of uncertainty, or the level of confidence that can be attached to these findings. In a few studies, panel members have been asked to nominate a confidence score (e.g. 0–3), to reflect their confidence in the scores produced for each technique assessed (Beausoleil & Mellor 2015; Beausoleil *et al.* 2016; Harvey *et al.* 2020; Baker *et al.* 2022). However, it is not always transparent how these confidence scores are arrived at, and many studies make no attempt to estimate uncertainty (Table 1).

The developers of the model have suggested that accessing scientifically informed expert opinion should minimise this uncertainty, but this has not been tested. Consequently, the outputs of the model may suggest a certain level of precision, but this is not calculated and so should be interpreted with caution (Mellor & Beausoleil 2015). Baker *et al.* (2016) recognised this limitation in their assessment of wildlife control methods, noting that “some rankings appeared counter-intuitive, highlighting the need for objective formal welfare assessments.”

Another source of uncertainty is derived from the fact that Five Domains assessments often consider procedural documents stating how practices *should be* conducted (Baker *et al.* 2016), rather than considering animal-based data documenting how they *are* conducted (Hampton *et al.* 2016b), including how often adverse events occur (Hampton *et al.* 2019). In general, this approach has taken the form of checklist audits assessing compliance with conditions prescribed in procedural documents to allow simple reporting to stakeholders. Hence, there is substantial uncertainty around whether the procedures that are assessed via Five Domains assessments are actually those that are performed.

### Unintentional and indirect impacts

As the Five Domains model was conceived for a laboratory context, it focuses on animals intentionally impacted by human activities (Mellor & Reid 1994). This works well enough for controlled environments such as laboratories. However, focus on a single species of animal becomes limiting in complex environments that contain multiple groups of animals (Fisher *et al.* 2019). In complex ecosystems, any single-species assessment excludes a large suite of processes that harm animals either unintentionally or indirectly (Hampton *et al.* 2022). For free-ranging animals, intentional and direct impacts on animals constitute only a fraction of the ways in which human activities harm animals (Fraser & MacRae 2011). After all, most of the ways in which humans harm animals are not intentional or even direct. In fact, most are derived from processes of which we may not be fully aware, such as the impacts of windows on wild birds (Loss et al. 2015) or extreme heat on wild bats such as grey-headed flying-foxes (*Pteropus poliocephalus*) (Mo *et al.* 2021). This realisation has led to the idea of ‘One Welfare’ (Pinillos *et al.* 2016).

There is no current pathway for incorporating these processes into Five Domains outputs, or at least there has been no attempt to do so thus far. We found no published studies that accounted for these effects (Table 1). It is particularly unfortunate that the Five Domains has been so widely used for wildlife, where unintentional and indirect effects are so impactful (Fraser & MacRae 2011; Allen & Hampton 2020; Hampton *et al.* 2021). Nonetheless, claims have been made that the model allows holistic animal welfare assessments for wildlife contexts (Sharp & Saunders 2011), but yet fail to account for processes that can profoundly contribute to the suffering of vast numbers of animals.

One example is assessment of kangaroo (*Macropus* and *Osphranter* spp) management options (Stephens 2021) that fails to account for poisoning of wildlife scavengers (Hampton *et al.* 2022). Similarly, wild rodent control assessments have been conducted (De Ruyver *et al.* 2023) without considering secondary poisoning of non-target wildlife species from anticoagulant rodenticides (Fisher *et al.* 2019). We acknowledge that failure to account for unintentional harms is shared by many proposed assessment tools that were designed for domestic animal contexts – this is not a weakness unique to the Five Domains.

### Misuse

We have concerns that the outputs of the Five Domains model may be communicated in a misleading way if they are presented as the product of a fully-fledged scientific assessment method. We are further concerned that this misrepresentation may progress to misuse (or even abuse) under certain circumstances. Due to the reliance on ‘expert’ opinion, the model is susceptible to manipulation by panel members with aligned professional interests advancing their own agendas by reaching a pre-conceived conclusion when assessing contentious practices. This may occur if panel members are hand-selected, and findings are communicated in opaque ways. Regrettably, the list of studies to have misused the model and/or failed to appropriately recognise limitations with model use includes some of the authors of this article (Hampton *et al.* 2016a; Allen *et al.* 2019).

At worst, the Five Domains assessment approach is highly manipulable if investigators wish to collude to reach a pre-agreed conclusion. In the very worst-case scenario, the outputs of a Five Domains assessment may amount to nothing more than the opinions of the loudest or most determined member of an opaquely selected panel, expressed as numerical scores without measures of uncertainty.

### Overview of limitations

The limitations listed and discussed above are certainly not unknown (Beausoleil & Mellor 2015). We do not wish to imply that we are the first to identify them. They have been explicitly acknowledged by the developers of the model, who have suggested that the model helpfully advances the evaluation of animal welfare impacts, provided that its limitations are borne in mind (Beausoleil & Mellor 2015; Mellor 2017; Mellor *et al.* 2020). However, we observe that, increasingly, researchers applying the Five Domains model are *not* bearing these critical limitations (e.g. subjective experiences to objective scores, expert opinion, repeatability, aggregation, uncertainty, and unintentional and indirect impacts) in mind.

Failure to acknowledge the limitations of the Five Domains model gives rise to the representation of the model as a ‘one-stop shop’ for animal welfare considerations in some contexts. We are concerned that opportunistic organisations may use the Five Domains model as a public relations façade (Hampton *et al.* 2016b). This strategy has allowed claims that animal welfare concerns have been assessed or addressed without anything more than desktop exercises being undertaken. However, we feel that there are some achievable steps that could be taken to improve the scientific validity of the Five Domains assessment approach.

### Suggestions for refinement

If the Five Domains continues to be used as an animal welfare measurement tool, there are several refinements that are necessary for it to become fit for this purpose. Like other such models, it will have its limitations which should be clearly stated. To enable refinement and transparency, a number of improvements should and could be made in the way that the model is used.

Firstly, studies are urgently needed to explore repeatability. There is a substantial body of literature on measuring repeatability and reducing variability in qualitative panel assessments (Vaz *et al.* 2013; Friedrich *et al.* 2020). Such studies could assess: (1) whether panels with different membership give the same scores to the same techniques assessed; (2) whether panel size influences scores; and (3) whether the same panel gives the same score when assessed at different time-points, and so forth.

Second, there is an onus on authors using the model to improve the transparency of their research by disclosing (at the minimum): (1) how their panel members were selected; (2) the size of their panel; (3) an overview of the literature provided to the panel; and (4) the process(es) used for resolving disagreement and reaching consensus.

Third, as shown by the study of De Ruyver *et al.* (2023), there is need for refinement (or at least transparency) in processes used to reach group-based final scores that either involve anonymous or confidential scoring in a democratic system or discussion/consensus. An alternative approach to those proposed previously may be a moderated discussion that culminates in anonymous scoring. The Delphi method uses a similar approach, whereby the vital elements include anonymity, controlled feedback, and iteration to refine stated opinions and reach consensus (Nasa *et al.* 2021).

Fourth, improved methods are required to translate subjective assessments into numerical scores, including: (1) whether the scores *within* domains can be assumed to be linearly related, e.g. does a score of 4 represent twice the impact as a score of 2, just as a score of 2 represents twice the impact as a score of 1?; and (2) how scores are aggregated *between* domains, accounting for what weighting is given to each domain. We acknowledge that there will never be a perfect solution to the problem of converting and summating numerical scores from subjective assessments. Each solution will be based on ethical and methodological assumptions that can be debated (Sandøe *et al.* 2019). However, what can be achieved is a solution where these assumptions are made transparent. Unlike what is the case now where conclusions about net welfare outcomes are drawn based on the Five Domains model in an opaque way.

Fifth, the way in which scientific uncertainty is estimated and communicated needs to be refined and made consistent. A simple approach to partially offset this problem is to convene two panels and compare their conclusions, or to publish and discuss the variability in the experts’ scores (see, for example, Sandøe *et al.* [2022] for a way to do this).

Sixth, unintentional and indirect impacts should be accounted for, particularly for wildlife studies, as per the ‘harms’ model (Fraser & MacRae 2011).

Seventh, there is a need for better, well-validated, animal-based measures for many species and management contexts (Harvey *et al.* 2023).

### Comparison to alternative approaches

There are several alternative animal welfare assessment approaches currently used that may be compared to the Five Domains model; each has its own strengths and weaknesses. Two notable examples are Welfare Quality® (Friedrich *et al.* 2020) and the Benchmark method (Sandøe *et al.* 2022). In contrast to the Five Domains, complex aggregation algorithms and repeatability measures have been developed for Welfare Quality® (Friedrich *et al.* 2020). The group behind the Welfare Quality® framework have developed a mathematical model to handle the various concerns relating to aggregation, notably to avoid positive values on some parameters being used to compensate for negative values on other parameters (Botreau *et al.* 2007a,b; Veissier *et al.* 2011). The group behind the Benchmark approach has developed a simpler approach where experts first value different conditions belonging to different domains and subsequently weigh the relative importance of the domains (Sandøe *et al.* 2022). However, many of the problems described above relating to selection of panel members may also apply to approaches such as Welfare Quality® and the Benchmark approach as well.

The ‘harms’ model (Fraser & MacRae 2011) is a useful way to visualise the ecosystem-wide consequences of any anthropogenic activity by systematically assessing its negative animal welfare impacts. The model was designed to explicitly include consideration of processes that harm animals but may not be perpetrated deliberately or widely known, sometimes referred to as ‘invisible’ harms (Finn & Stephens 2017). However, this model generates only a list of harmful processes, with no attempts made at grading severity or attempting aggregation. It is useful for visualising the *breadth* of animal welfare impacts associated with any activity (Hampton *et al.* 2021) and this facet could potentially be incorporated into Five Domains assessments if there was a desire to make assessments truly holistic.

## Why has this not been challenged before?

Given some of the concerns we have identified above relating to the use of the Five Domains model as a scientific assessment tool, it is reasonable to ask why this has not been challenged more often (or at all) over recent decades. We can only speculate here.

The model is intuitive, relying largely upon its simplicity: any animal manipulation practice of interest can be assessed by sitting down with a few colleagues in a room for a day. In many cases, a publishable paper can be produced from these efforts, which can then be cited by others as ‘evidence’ that the assessment method is sound. Use of the model is undoubtedly convenient. It is quick and inexpensive to use and allows simple reporting to stakeholders. It allows animal welfare researchers to publish supposedly science-based assessments from the desktop without the inconvenience of those logistical elements required for animal-based research, including licences from institutional animal research committees.

The appeal for industry bodies is understandable too, given the costs of commissioning a Five Domains panel will be a fraction of that for a multi-year animal experiment or monitoring programme. The results can also be controlled more easily than independent research that produces incontrovertible results, through selection of panel members or consensus/democracy processes. But we contend that this convenience comes at a price, and scientific legitimacy is the most important price that the animal welfare community may pay. However, we are not suggesting that the majority of practitioners resorting to Five Domains assessments do so because they have ulterior motives or are lax, but because they are trying to weigh up welfare challenges that are currently difficult to compare empirically using the tools of science. Nonetheless, the approach remains concerningly susceptible to manipulation and misuse.

### Animal welfare implications

For animal welfare to maintain scientific legitimacy, it is essential that the most objective and transparent methods (recognising that there are likely to be several competing models) are used to empirically investigate how animals are affected by human interventions and activities. This legitimacy will undoubtedly be eroded if animal welfare publications are seen to amount to nothing more than assertions of eminence from groups that include community members or stakeholders who may lack an understanding of animal-based evidence and its welfare implications, and whose impartiality may be called into question. We contend that the Five Domains model is appropriate for conceptualising animal welfare impacts and enhancements. It also seems reasonable to use this qualitative tool to highlight areas of welfare risk, as well as areas requiring further investigation, as proposed by Sherwen *et al.* (2018). It could also be used as a “hypothesis generating” (Biesecker 2013) device, whereby the conclusions from a Five Domains panel are adapted into a hypothesis relating to potential welfare impacts that may be occurring, which can then be explored using scientific methods (Baker *et al.* 2016). However, even in this context, limitations with use of the models do need to be appropriately recognised. In our view, the Five Domains model is not currently developed into a suitable method for quantifying or ranking animal welfare impacts. The problems we have outlined with this approach cannot be ignored. We suggest that using the outputs of the model, in its current state, to guide policy in the guise of a science-based approach is particularly fraught (Johnson *et al.* 2019). We think the model could be developed into a more credible and useful assessment tool if some of our above suggestions for improvements are followed and if results are reported in a way where there is full transparency about their limitations.

## Conclusion

In conclusion, we want to be clear that we see great value in the Five Domains model as a way of *thinking about* animal welfare. It has undoubtedly made a substantial improvement on earlier influential paradigms in animal welfare such as the Five Freedoms. It is a wonderful *starting point* for animal welfare conversations and is fit to serve as the nucleus for decision-making processes. However, like the Five Freedoms paradigm before it (McCulloch 2013), the model is not currently fit-for-purpose as an assessment tool. We contend that some attempts to utilise it as such a tool, especially in poorly studied contexts, misuse the model, and may stifle empirical animal-based studies (Hampton *et al.* 2016b). To be clear, the limitations we have outlined here in varying degrees apply to other conceptual frameworks when used as an assessment tool. We perceive a reputational risk if animal welfare scientists knowingly continue to use an approach that is not transparent nor repeatable. If this trend is not arrested, we feel that there is a very real risk that animal welfare will increasingly be viewed as a pseudoscience (a practice mistakenly regarded as being based on scientific methods) (Truran 2013) by other stakeholders. After all, a key marker of scientific claims is that they are testable and therefore falsifiable. We look forward to seeing use of the Five Domains in its current form developed by the next generation of animal welfare scientists. If nothing else, we hope that this paper stimulates further discussion and some re-thinking or revision of the use of the Five Domains as an integrated assessment of animal welfare.

## References

[r1] Allen BL, Allen LR, Ballard G, Drouilly M, Fleming PJ, Hampton JO, Hayward MW, Kerley GI, Meek PD and Minnie L 2019 Animal welfare considerations for using large carnivores and guardian dogs as vertebrate biocontrol tools against other animals. Biological Conservation 232: 258–270. 10.1016/j.biocon.2019.02.019

[r2] Allen BL and Hampton JO 2020 Minimizing animal welfare harms associated with predation management in agro‐ecosystems. Biological Reviews 95: 1097–1108. 10.1111/brv.12601 32302055

[r3] Appleby MC and Sandøe P 2002 Philosophical debate on the nature of well-being: implications for animal welfare. Animal Welfare 11(3): 283–294. 10.1017/S0962728600024866

[r4] Baker S, Ayers M, Beausoleil N, Belmain S, Berdoy M, Buckle A, Cagienard C, Cowan D, Fearn-Daglish J, Goddard P, Golledge HDR, Mullineaux E, Sharp T, Simmons A and Schmolz E 2022 An assessment of animal welfare impacts in wild Norway rat (*Rattus norvegicus*) management. Animal Welfare 31: 51–68. 10.7120/09627286.31.1.005

[r5] Baker SE, Sharp TM and Macdonald DW 2016 Assessing animal welfare impacts in the management of European rabbits (*Oryctolagus cuniculus*), European moles (*Talpa europaea*) and carrion crows (*Corvus corone*). *PloS ONE* 11: e0146298. 10.1371/journal.pone.0146298 PMC4699632 26726808

[r6] Beausoleil N, Fisher P, Littin K, Warburton B, Mellor D, Dalefield R and Cowan P 2016 A systematic approach to evaluating and ranking the relative animal welfare impacts of wildlife control methods: poisons used for lethal control of brushtail possums (*Trichosurus vulpecula*) in New Zealand. Wildlife Research 43: 553–565. 10.1071/WR16041

[r7] Beausoleil N and Mellor D 2015 Advantages and limitations of the Five Domains model for assessing welfare impacts associated with vertebrate pest control. New Zealand Veterinary Journal 63: 37−43. 10.1080/00480169.2014.956832 25147947

[r8] Beausoleil NJ, Baker SE and Sharp T 2022 Scientific assessment of the welfare of trapped mammals—key considerations for the use of the Sharp and Saunders humaneness assessment model. Animals 12: 402. 10.3390/ani12030402 35158725 PMC8833337

[r9] Beausoleil NJ, Mellor DJ, Baker L, Baker SE, Bellio M, Clarke AS, Dale A, Garlick S, Jones B and Harvey A 2018 “Feelings and fitness” not “feelings or fitness”–the raison d’être of conservation welfare, which aligns conservation and animal welfare objectives. Frontiers in Veterinary Science 5: 296. 10.3389/fvets.2018.00296 30538995 PMC6277474

[r10] Biesecker LG 2013 Hypothesis-generating research and predictive medicine. Genome Research 23: 1051–1053. 10.1101/gr.157826.113 23817045 PMC3698497

[r11] Botreau R, Bonde M, Butterworth A, Perny P, Bracke M, Capdeville J and Veissier I 2007a Aggregation of measures to produce an overall assessment of animal welfare. Part 1: a review of existing methods. Animal 1: 1179–1187. 10.1017/S1751731107000535 22444862

[r12] Botreau R, Bracke M, Perny P, Butterworth A, Capdeville J, Van Reenen C and Veissier I 2007b Aggregation of measures to produce an overall assessment of animal welfare. Part 2: analysis of constraints. Animal 1: 1188–1197. 10.1017/S1751731107000547 22444863

[r13] Boys RM, Beausoleil NJ, Pawley MD, Betty EL and Stockin KA 2022a Evaluating potential cetacean welfare indicators from video of live stranded long-finned pilot whales (*Globicephala melas edwardii*). Animals 12(14): 1861. 10.3390/ani12141861 35883407 PMC9312325

[r14] Boys RM, Beausoleil NJ, Pawley MD, Littlewood KE, Betty EL and Stockin KA 2022b Fundamental concepts, knowledge gaps and key concerns relating to welfare and survival of stranded cetaceans. Diversity 14: 338. 10.3390/d14050338

[r15] Brambell FWR 1965 Report of the Technical Committee to Enquire into the Welfare of Animals Kept Under Intensive Livestock Husbandry Systems. Her Majesty’s Stationery Office: London, UK.

[r16] Broom DM 2011 A history of animal welfare science. Acta Biotheoretica 59: 121–137. 10.1007/s10441-011-9123-3 21347723

[r17] Browning H 2022a Assessing measures of animal welfare. Biology & Philosophy 37: 36. 10.1007/s10539-022-09862-1 PMC10008771 36926384

[r18] Browning H 2022b The measurability of subjective animal welfare. Journal of Consciousness Studies 29(3–4): 150–179. 10.53765/20512201.29.3.150

[r19] Buddle EA, Bray HJ and Ankeny RA 2018 Why would we believe them? Meat consumers’ reactions to online farm animal welfare activism in Australia. Communication Research and Practice 4: 246–260. 10.1080/22041451.2018.1451209

[r20] Clegg IL, Borger-Turner JL and Eskelinen HC 2015 C-Well: The development of a welfare assessment index for captive bottlenose dolphins (*Tursiops truncatus*). Animal Welfare 24(3): 267–282. 10.7120/09627286.24.3.267

[r21] Cullen HJ and Monds LA 2020 Jury simulation studies: to exclude or not to exclude participants based on a lack of comprehension of the case? Applied Cognitive Psychology 34(5): 1224–1233. 10.1002/acp.3695

[r22] De Ruyver C, Baert K, Cartuyvels E, Beernaert L, Tuyttens F, Leirs H and Moons C 2023 Assessing animal welfare impact of fourteen control and dispatch methods for house mouse (*Mus musculus*), Norway rat (Rattus norvegicus) *and black rat* (Rattus rattus). Animal Welfare 32: 1–10. 10.1017/awf.2022.2 PMC10937213 38487454

[r23] EFSA Panel on Animal Health and Welfare (AHAW) 2012 Guidance on risk assessment for animal welfare. EFSA Journal 10(1): 2513. 10.2903/j.efsa.2012.2513

[r24] Farm Animal Welfare Committee 1979 Five Freedoms. Farm Animal Welfare Council: London, UK.

[r25] Finn H and Stephens N 2017 The invisible harm: land clearing is an issue of animal welfare. Wildlife Research 44: 377–391. 10.1071/WR17018

[r26] Fisher P, Campbell KJ, Howald GR and Warburton B 2019 Anticoagulant rodenticides, islands, and animal welfare accountancy. Animals 9(11): 919. 10.3390/ani9110919 31690063 PMC6912481

[r27] Fletcher K, Cameron L and Freeman M 2021 Contemplating the Five Domains model of animal welfare assessment: UK horse owner perceptions of equine well-being. Animal Welfare 30: 259–268. 10.7120/09627286.30.3.003

[r28] Fogarty E, Swain D, Cronin G and Trotter M 2019 A systematic review of the potential uses of on-animal sensors to monitor the welfare of sheep evaluated using the Five Domains Model as a framework. Animal Welfare 28: 407–420. 10.7120/09627286.28.4.407

[r29] Fraser D 2008 Understanding animal welfare. Acta Veterinaria Scandinavica 50: S1. 10.1186/1751-0147-50-S1-S1 19049678 PMC4235121

[r30] Fraser D and Duncan IJ 1998 ‘Pleasures’,‘pains’ and animal welfare: toward a natural history of affect. Animal Welfare 7(4): 383–396. 10.1017/S0962728600020935

[r31] Fraser D and MacRae AM 2011 Four types of activities that affect animals: Implications for animal welfare science and animal ethics philosophy. Animal Welfare 20: 581–590. 10.1017/S0962728600003213

[r32] Friedrich L, Krieter J, Kemper N and Czycholl I 2020 Interobserver reliability of measures of the Welfare Quality® animal welfare assessment protocol for sows and piglets. Animal Welfare 29(3): 323–337. 10.7120/09627286.29.3.323

[r33] Gavrilets S, Auerbach J and Van Vugt M 2016 Convergence to consensus in heterogeneous groups and the emergence of informal leadership. Scientific Reports 6: 29704. 10.1038/srep29704 27412692 PMC4944200

[r34] Grandin T 2022 Practical application of the Five Domains animal welfare framework for supply food animal chain managers. Animals 12(20): 2831. 10.3390/ani12202831 36290216 PMC9597751

[r35] Hampton JO, Hyndman TH, Allen BL and Fischer B 2021 Animal harms and food production: informing ethical choices. Animals 11(5): 1225. 10.3390/ani11051225 33922738 PMC8146968

[r36] Hampton JO, Hyndman TH, Laurence M, Perry AL, Adams P and Collins T 2016b Animal welfare and the use of procedural documents: limitations and refinement. Wildlife Research 43(7): 599–603. 10.1071/WR16153

[r37] Hampton JO, Jones B, Perry AL, Miller CJ and Hart Q 2016a Integrating animal welfare into wild herbivore management: lessons from the Australian Feral Camel Management Project. The Rangeland Journal 38(2): 163–171. 10.1071/RJ15079

[r38] Hampton JO, MacKenzie DI and Forsyth DM 2019 How many to sample? Statistical guidelines for monitoring animal welfare outcomes. PloS ONE 14(1): e0211417. 10.1371/journal.pone.0211417 PMC6353194 30699193

[r39] Hampton JO, Pay JM, Katzner TE, Arnemo JM, Pokras MA, Buenz E, Kanstrup N, Thomas VG, Uhart M, Lambertucci SA, Krone O, Singh NJ, Naidoo V, Ishizuka M, Saito K, Helander B and Green RE 2022 Improving the management of macropods without poisoning ecosystems. Ecological Management & Restoration 23(2): 153–157. 10.1111/emr.12555

[r40] Harley JJ, Stack JD, Braid H, McLennan KM and Stanley CR 2021 Evaluation of the feasibility, reliability, and repeatability of welfare indicators in free-roaming horses: a pilot study. Animals 11(7): 1981. 10.3390/ani11071981 34359108 PMC8300213

[r41] Harvey AM, Beausoleil NJ, Ramp D, and Mellor DJ 2023 Mental experiences in wild animals: scientifically validating measurable welfare indicators in free-roaming horses. Animals 13(9): 1507. 10.3390/ani13091507 37174544 PMC10177449

[r42] Harvey AM, Beausoleil NJ, Ramp D and Mellor DJ 2020 A ten-stage protocol for assessing the welfare of individual non-captive wild animals: free-roaming horses (*Equus ferus caballus*) as an example. Animals 10(1): 148. 10.3390/ani10010148 31963232 PMC7022444

[r43] Harvey AM, Morton JM, Mellor DJ, Russell V, Chapple RS and Ramp D 2021 Use of remote camera traps to evaluate animal-based welfare indicators in individual free-roaming wild horses. Animals 11(7): 2101. 10.3390/ani11072101 34359229 PMC8300222

[r44] Harvey AM, Ramp D and Mellor DJ 2022 Review of the foundational knowledge required for assessing horse welfare. Animals 12(23): 3385. 10.3390/ani12233385 36496906 PMC9736110

[r45] Hernandez RO, Sánchez JA and Romero MH 2020 Iceberg indicators for animal welfare in rural sheep farms using the five domains model approach. Animals 10(12): 2273. 10.3390/ani10122273 33276493 PMC7760869

[r46] Johnson AK, Rault J-L, Marchant JN, Baxter EM and O’Driscoll K 2022 Improving young pig welfare on-farm: the Five Domains Model. Journal of Animal Science 100(6): skac164. 10.1093/jas/skac164 PMC9202571 35536191

[r47] Johnson CN, van Bommel L and Williams D 2019 Livestock guardian dogs and animal welfare: Comment on Allen et al. (2019) ‘Animal welfare considerations for using large carnivores and guardian dogs as vertebrate biocontrol tools against other animals’. Biological Conservation 236: 580–581. 10.1016/j.biocon.2019.04.017

[r48] Kagan R, Carter S and Allard S 2015 A universal animal welfare framework for zoos. Journal of Applied Animal Welfare Science 18: S1–S10. 10.1080/10888705.2015.1075830 26440493 PMC4673521

[r49] Kells N 2021 The Five Domains model and promoting positive welfare in pigs. Animal 16(2): 100378. 10.1016/j.animal.2021.100378 34697006

[r50] Ledger RA and Mellor DJ 2018 Forensic use of the five domains model for assessing suffering in cases of animal cruelty. Animals 8: 101. 10.3390/ani8070101 29941781 PMC6071132

[r51] Littlewood KE and Mellor DJ 2016 Changes in the welfare of an injured working farm dog assessed using the Five Domains Model. Animals 6: 58. 10.3390/ani6090058 27657140 PMC5035953

[r52] Loss SR, Will T and Marra PP 2015 Direct mortality of birds from anthropogenic causes. Annual Review of Ecology, Evolution, and Systematics 46: 99–120. 10.1146/annurev-ecolsys-112414-054133

[r53] McCulloch SP 2013 A critique of FAWC’s five freedoms as a framework for the analysis of animal welfare. Journal of Agricultural and Environmental Ethics 26(5): 959–975. 10.1007/s10806-012-9434-7

[r54] McGreevy P, Berger J, De Brauwere N, Doherty O, Harrison A, Fiedler J, Jones C, McDonnell S, McLean A and Nakonechny L 2018 Using the five domains model to assess the adverse impacts of husbandry, veterinary, and equitation interventions on horse welfare. Animals 8(3): 41. 10.3390/ani8030041 29562654 PMC5867529

[r55] Mellor D and Beausoleil N 2015 Extending the ‘Five Domains’ model for animal welfare assessment to incorporate positive welfare states. Animal Welfare 24(3): 241−253. 10.7120/09627286.24.3.241

[r56] Mellor D and Burns M 2020 Using the Five Domains Model to develop welfare assessment guidelines for Thoroughbred horses in New Zealand. New Zealand Veterinary Journal 68(3): 150–156. 10.1080/00480169.2020.1715900 31973682

[r57] Mellor D and Reid C 1994 Concepts of animal well-being and predicting the impact of procedures on experimental animals, In: Baker R, Jenkin G and Mellor D (Eds.) Improving the Well-being of Animals in the Research Environment: *pp 3−18 Australian and New Zealand Council for the Care of Animals in Research and Teaching*: Sydney, Australia.

[r58] Mellor DJ 2016 Updating animal welfare thinking: moving beyond the “Five Freedoms” towards “a Life Worth Living”. Animals 6(3): 21. 10.3390/ani6030021 27102171 PMC4810049

[r59] Mellor DJ 2017 Operational details of the five domains model and its key applications to the assessment and management of animal welfare. Animals 7(8): 60. 10.3390/ani7080060 28792485 PMC5575572

[r60] Mellor DJ, Beausoleil NJ, Littlewood KE, McLean AN, McGreevy PD Jones B, and Wilkins C 2020 The 2020 five domains model: including human–animal interactions in assessments of animal welfare. Animals 10(10): 1870. 10.3390/ani10101870 33066335 PMC7602120

[r61] Mellor DJ, Hunt S and Gusset M 2015 Caring for Wildlife: the World Zoo and Aquarium Animal Welfare Strategy. World Association of Zoos and Aquariums: Gland, Switzerland.

[r62] Mo M, Roache M, Davies J, Hopper J, Pitty H, Foster N, Guy S, Parry-Jones K, Francis G and Koosmen A 2021 Estimating flying-fox mortality associated with abandonments of pups and extreme heat events during the austral summer of 2019–20. Pacific Conservation Biology 28: 124–139. 10.1071/PC21003

[r63] Nasa P, Jain R and Juneja D 2021 Delphi methodology in healthcare research: how to decide its appropriateness. World Journal of Methodology 11(4): 116–129. 10.5662/wjm.v11.i4.116 34322364 PMC8299905

[r64] Nickerson RS 1998 Confirmation bias: a ubiquitous phenomenon in many guises. Review of General Psychology 2(2): 175–220. 10.1037/1089-2680.2.2.175

[r65] Normyle A, Vardon M Doran B 2022 Ecosystem accounting and the need to recognise Indigenous perspectives. Humanities and Social Sciences Communications 9:133. 10.1057/s41599-022-01149-w

[r66] O’Callaghan K, Cripps P, Downham D and Murray R 2003 Subjective and objective assessment of pain and discomfort due to lameness in dairy cattle. Animal Welfare 12(4): 605–610. 10.1017/S0962728600026257

[r67] Park M, Leahey E, and Funk RJ 2023 Papers and patents are becoming less disruptive over time. Nature 613(7942): 138−144. 10.1038/s41586-022-05543-x 36600070

[r68] Pinillos RG, Appleby MC, Manteca X, Scott-Park F, Smith C and Velarde A 2016 One Welfare - a platform for improving human and animal welfare. Veterinary Record 179(16): 412–413. 10.1136/vr.i5470 27770094

[r69] Resnik DB, and Smith EM 2020 Bias and groupthink in science’s peer-review system, In: Allen DM and Howell JW (Eds.) Groupthink in Science pp 99–113 Springer: New York City, USA. 10.1007/978-3-030-36822-7_9

[r70] Sandøe P, Corr SA, Lund TB and Forkman B 2019 Aggregating animal welfare indicators: can it be done in a transparent and ethically robust way? Animal Welfare 28(1): 67–76. 10.7120/09627286.28.1.067

[r71] Sandøe P, Hansen HO, Forkman B, van Horne P, Houe H, de Jong IC, Kjær JB, Nielsen SS, Palmer C, Rhode HLH, and Christensen T 2022 Market driven initiatives can improve broiler welfare–a comparison across five European countries based on the Benchmark method. Poultry Science 101(5): 101806. 10.1016/j.psj.2022.101806 PMC8965143 35349952

[r72] Sharp T and Saunders G 2011 *A Model Ffor Assessing the Relative Humaneness of Pest Animal Control Methods* . Department of Agriculture, Fisheries and Forestry: Canberra, Australia.

[r73] Sherwen SL, Hemsworth LM, Beausoleil NJ, Embury A and Mellor DJ 2018 An animal welfare risk assessment process for zoos. Animals 8(8): 130. 10.3390/ani8080130 30060544 PMC6116011

[r74] Shpungin E, Allen N, Loomis C and DelloStritto ME 2012 Keeping the spirit alive: using feminist methodology to address silencing. Journal of Community Psychology 40(1): 44–61. 10.1002/jcop.20481

[r75] Stephens T 2021 Kangaroo management and animal welfare. Ecological Management and Restoration 22: 71–74. 10.1111/emr.12469

[r76] Truran P 2013 Practical Applications of the Philosophy of Science: Thinking about Research. Springer: Cham, Switzerland. 10.1007/978-3-319-00452-5

[r77] Van der Schot AA and Phillips C 2013 Publication bias in animal welfare scientific literature. Journal of Agricultural and Environmental Ethics 26(5): 945–958. 10.1007/s10806-012-9433-8

[r78] Vaz S, Falkmer T, Passmore AE, Parsons R and Andreou P 2013 The case for using the repeatability coefficient when calculating test–retest reliability. PloS ONE 8(9): e73990. 10.1371/journal.pone.0073990 PMC3767825 24040139

[r79] Veissier I, Jensen KK, Botrea R and Sandøe P 2011 Highlighting ethical decisions underlying the scoring of animal welfare in the Welfare Quality® scheme. Animal Welfare 20(1): 89. 10.1017/S0962728600002463

[r80] Ward SJ, Williams E, Groves G, Marsh S and Morgan D 2020 Using zoo welfare assessments to identify common issues in developing country zoos. Animals 10(11): 2101. 10.3390/ani10112101 33198237 PMC7696472

[r81] Windschnurer I, Schmied C, Boivin X and Waiblinger S 2008 Reliability and inter-test relationship of tests for on-farm assessment of dairy cows’ relationship to humans. Applied Animal Behaviour Science 114(1–2): 37–53. 10.1016/j.applanim.2008.01.017

